# Current landscape and future directions of policies addressing air quality improvement in Pakistan: a scoping review

**DOI:** 10.7189/jogh.15.04349

**Published:** 2025-12-29

**Authors:** Maham Zahid, Ramsha Tariq Baig, Hareem Fatima, Hana Mahmood, Paras Shakeel, Amina Khan, Genevie Fernandes, Sajid Soofi, Osman Mohammad Yusuf, Shabina Ariff, Linda Bauld, RESPIRE collaboration

**Affiliations:** 1 The Initiative, Islamabad, Pakistan; 2 The Allergy and Asthma Institute, Islamabad, Pakistan; 3 Aga Khan University, Karachi, Pakistan; 4 Neoventive Solutions, Islamabad, Pakistan; 5 Usher Institute, University of Edinburgh, Edinburgh, UK

## Abstract

**Background:**

Pakistan ranks third in global air-pollution burden, yet evidence on how its air quality-related policies are implemented remains fragmented. We aim to map the existing air quality policies in Pakistan, identify barriers to policy implementation, and highlight policy priorities for improving air quality governance.

**Methods:**

We conducted a scoping review following Arksey-O’Malley and PRISMA-ScR guidelines to map national and provincial air-quality policies, describe their implementation, and identify barriers, facilitators and priority actions. Searches of PubMed, Scopus, and grey literature sources to January 2024 yielded 1438 records; 27 documents (eight peer-reviewed articles, 19 policy reports) met the inclusion criteria. We charted the data on policy characteristics, implementation status, and contextual factors. We synthesised the findings using a narrative descriptive approach.

**Results:**

Seven federal and nine provincial instruments address air quality, including the Pakistan Environmental Protection Act (1997), National Clean Air Policy (2023), and four provincial clean air action plans. Devolved governance, weak enforcement capacity, limited monitoring networks, and scarce, decentralised emissions data hamper implementation. Industrial and transport emissions dominate, while household solid-fuel use and open crop burning persist. Facilitators include recent adoption of Euro-V/VI fuel standards, growing citizen-science monitoring, and policy alignment with climate-change agendas. Priority actions are: a legally mandated federal-provincial task force; an integrated national emissions database combining ground and satellite data; incentive-based regulation with clear standards and fiscal levers; and public engagement through education, media, and community monitoring.

**Conclusions:**

Pakistan has a range of air quality-related policies but lacks the coordinated governance, data infrastructure, and market incentives needed to translate its intent into cleaner air. Bridging these gaps is essential to avert a mounting public health crisis.

**Registration:**

Open Science Foundation (https://doi.org/10.17605/OSF.IO/6ZAE9).

Air quality refers to the cleanliness of the air, whereas air pollution is the presence of harmful chemical, physical, or biological substances in the atmosphere [1]. Common pollutants include particulate matter (PM), nitrogen oxides, ozone, sulphur oxides, carbon monoxide (CO), and lead. Major sources of air pollution include motor vehicle emissions, industrial processes, household combustion, and forest fires. PM10 and PM2.5 – airborne particles with diameters of 10 and 2.5 μm or smaller, respectively – serve as key proxy measures of air quality [2]. The World Health Organization (WHO) recommends annual average concentrations not exceeding 5 μg/m^3^ for PM2.5 and 15 μg/m^3^ for PM10 as global standards for clean air [3].

Ozone, PM, nitrogen dioxide, sulphur dioxide (SO2), and CO are among the pollutants that pose a serious threat to public health [4]. Both indoor and outdoor air pollution are major contributors to morbidity and mortality from respiratory diseases (asthma, bronchitis), cardiovascular conditions (ischaemia), and cancers [5,6]. The WHO estimates seven million preventable deaths annually from outdoor and indoor air pollution [7]. Elevated pollution levels are also associated with cognitive decline, mental health disorders, and increased healthcare costs [8]. An increase of 10 μg/m^3^ in PM2.5 concentration is estimated to reduce life expectancy by nearly one year [9].

In Pakistan, global climate change and deteriorating urban air quality have emerged as significant environmental and public health challenges [10]. Over the past five years, air quality in many Pakistani cities has declined markedly, with cities like Lahore, Karachi, and Faisalabad frequently ranked among the most polluted globally.[11] Pakistan is the third-highest country in the world in terms of air pollution-related mortality, with PM2.5 concentrations consistently exceeding WHO guidelines [12]. Key contributors to poor air quality include emissions from transportation, industrial activity, agriculture, and household use of fossil fuels [13,14]. Despite gaps in air quality monitoring, there is a broad consensus that PM is the dominant pollutant in urban areas, followed by nitrogen oxides, SO2, CO, ozone, volatile organic compounds and lead [15]. This issue is exacerbated by the use of older, poorly maintained vehicles and chronic traffic congestion. Industrial emissions, particularly from brick kilns, steel mills, and cement factories, further exacerbate air pollution – especially in urban industrial hubs in Karachi, Lahore, and Faisalabad [16]. Agricultural practices also impact air quality, particularly during winter when crop residue burning significantly elevates pollution levels, particularly in the Punjab province [17]. Although limited research exists to quantify the contribution of each source, a 2018 World Food Organization survey estimated that transportation accounts for 43% of emissions, followed by industry (25%), agriculture (20%), and power generation (12%) [9]. Vehicular and industrial emissions together constitute the largest aggregate source of air pollution. According to available data, the average Pakistani loses 2.7 years of life due to air pollution, while residents of the province of Lahore lose up to 5.3 years [18,19].

Pakistan has enacted air pollution legislation at both national and provincial levels [20]. Following the 18th Constitutional Amendment in 2010, authority over key sectors – including health, agriculture, education, environment, and labour – was devolved to the four provinces: Punjab, Sindh, Baluchistan, and Khyber Pakhtunkhwa (KP) [21]. This decentralisation allowed provincial governments greater autonomy to legislate and manage environmental issues. However, despite policies aimed at improving air quality, evidence on their implementation remains limited, and there is a lack of comprehensive understanding of the factors that enable or hinder meaningful, sustained improvements nationwide.

Given the widespread impact of air pollution on health and development, synthesising available evidence and practical recommendations is essential for policymakers and practitioners. We aimed to fill this gap by mapping existing air quality policies in Pakistan, identifying barriers and enablers to policy implementation, and highlighting policy priorities and actionable recommendations for improving air quality governance at both the federal and provincial levels.

## METHODS

We conducted this scoping review as part of a larger project to identify and prioritise existing air quality policies in Pakistan, in collaboration with stakeholders through consultative workshops. Accordingly, we designed the review with a focused scope to identify and collate all available policy documents on air quality in Pakistan. Unlike broader scoping reviews that typically aim to map the entire body of evidence, our inclusion criteria were intentionally restricted to documents that explicitly address air quality**-**related policies to ensure alignment with the project’s objectives. We conducted the scoping review following the methodological framework outlined by Arksey and O’Malley [22]. We searched published scientific articles in online databases and grey literature from the internet and the websites of national organisations and regulatory authorities. We define policy as a set of guidelines that direct the actions of the government and serve as a strategic framework for decision-making, planning, and governance, while an act is a formal law with a legally binding nature, non-compliance with which can lead to penalties, fines, or legal consequences. For this review, ‘policies’ comprised all the laws, regulations, strategies and acts formulated and adopted by a governing body, including the federal or provincial government. Air quality-specific policies included those on air pollution and related areas, such as transport, vehicle emissions, industry, agriculture, climate change, power and energy, urban planning, and waste management. We reported this review in accordance with the PRISMA-ScR guidelines [23] and registered the protocol with the Open Science Foundation.

We identified grey literature through targeted searches of official government websites, international organi**s**ations, and policy repositories relevant to air quality in Pakistan. Two reviewers (MZ and HF) independently applied the same inclusion and exclusion criteria used for peer-reviewed studies. We first screened the documents by title and abstract or executive summary, and then by full text when available. Eligibility was confirmed for reports, guidelines, or policy documents that discussed or outlined air quality policies in Pakistan. We excluded papers that did not mention air quality policies, were unrelated in context, or were not publicly accessible. This systematic approach ensured that grey literature was reviewed and included in a transparent and reproducible manner.

For both peer-reviewed and grey literature, we charted the data on document type, issuing body, year of publication, and policy content. Where credible information was available from policy briefs, official reports, or government/organi**s**ational websites, we also noted the reported status of policy implementation. We descriptively recorded this information, which was not subjected to systematic evaluation or outcome analysis.

### Research question

What is known about policies developed and implemented to improve air quality in Pakistan? In doing so, we were interested in air quality policies, the extent and process of their implementation, any reported barriers and facilitators to achieving these policy goals, and whether any recommendations for improvements in policy implementation were made.

### Identifying relevant studies

We formulated search strings (Appendix S1 in the **Online Supplementary Document**) relevant to the research question by first listing search terms related to air quality, air pollution, policies, and the provinces/major cities of Pakistan, and then finalising the terms and operational definitions through consultations with policy stakeholders. An information specialist at Aga Khan University, Karachi, tested and vetted the search strings. We conducted preliminary searches in the PubMed, Medline, and Scopus databases using the finalised search strings to identify the scientific literature. In contrast, we used Google Scholar, Google, and OpenGrey to search the grey literature, including policy documents, policy reports/briefs, and websites of national agencies regulating air quality/environment control and international organisations (*e.g.* WHO, World Bank, and United Nations Environment Programme). We also explored websites of organisations and coalitions working on air quality, including the Pakistan Environmental Protection Agency, the Climate and Clean Air Coalition, the Pakistan Environment Trust, the Pakistan Clean Air Initiative, and the Asian Development Bank. Considering the lack of scientific literature from Pakistan on air quality policy implementation, we carefully searched grey literature sources to ensure that all relevant information was gathered. We also searched reference lists of included studies to ensure that no relevant studies were missed during screening. We uploaded the abstracts to Covidence for abstract, full-text, and data extraction.

### Study selection

All published articles and grey literature, including reports, guidelines, and policy documents in English, were eligible for inclusion if they addressed or discussed air quality policies in Pakistan. We also considered documents from official sites, including government policies, standards, or regulations concerning air quality. We systematically searched grey literature using advanced Google search operators (*e.g.* Boolean terms, site: queries), conducted targeted searches of federal and provincial government websites, institutional repositories, and snowballed from reference lists. We logged, screened, and manually deduplicated all retrieved records before applying the inclusion criteria. Given the absence of fixed definitions and the blurred distinctions between different policy document types in Pakistan, we adopted a broad approach by including all relevant legal and strategic documents, such as policies, acts, laws, action plans, and frameworks. We excluded literature if it did not include any information related to air quality policies in the abstract, if the full-text papers were not publicly available, or if they were from any country other than Pakistan. Two reviewers (MZ and HF) then applied the inclusion and exclusion criteria to all published citations; similarly, two additional reviewers (RTB and HM) used the same approach to grey literature.

**Figure 1:**
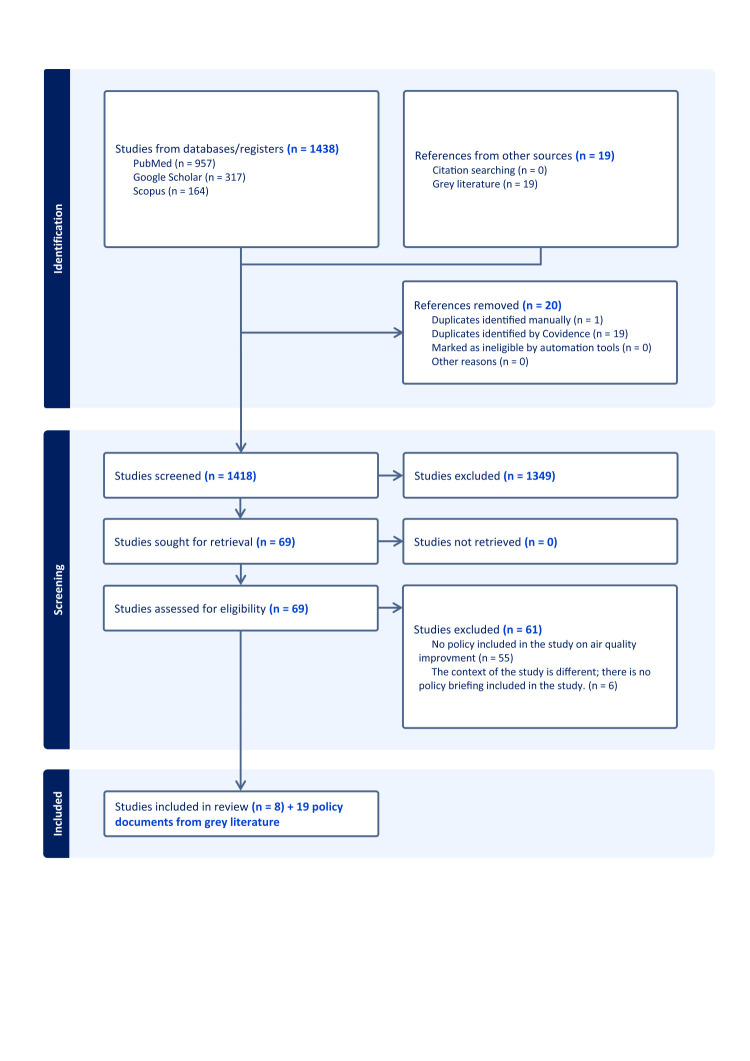
PRISMA flowchart.

We imported 1438 scientific study references for screening. During the initial phase, one duplicate was identified manually, and an additional 19 duplicates were identified by Covidence, resulting in 1418 studies screened based on title and abstract. Out of these, we excluded 1349 studies. Subsequently, we assessed 69 studies for full-text eligibility. Of these, we excluded 61 studies – 55 because they did not include any policy on air quality improvement, and six because the study context was unrelated and did not provide any policy briefing (**Figure 1**). As a result, we included eight studies in the final analysis, with no ongoing studies or studies awaiting classification. From grey literature, we included 19 policy documents and reports (**Table 1**).

**Table 1:** List of included literature

Author, reference	Year	Type of literature
Ahmed *et al.*, [24]	2023	Scientific paper
Colbeck *et al.*, [18]	2010	Scientific paper
Anjum *et al.*, [11]	2021	Scientific paper
Anwar *et al.*, [15]	2021	Scientific paper
Mir *et al.*, [25]	2016	Scientific paper
Mir *et al.*, [26]	2024	Scientific paper
Hussain *et al.*, [27]	2019	Scientific paper
Aziz *et al.*, [28]	2006	Scientific paper
Ministry of Climate Change, [29]	2023	Grey literature
Pakistan Environmental Protection Agency, [30]	2021	Grey literature
Ministry of Climate Change and Environmental Coordination, [31]	2005	Grey literature
Ministry of Climate Change and Environmental Coordination, [32]	2023	Grey literature
Ministry of Climate Change and Environmental Coordination, [33]	2021	Grey literature
Environment Protection Department, Government of the Punjab, [34]	2023	Grey literature
Asian Development Bank, [35]	2021	Grey literature
Environment Protection Department, Government of the Punjab, [36]	2017	Grey literature
Consortium for Development Policy Research, [37]	2019	Grey literature
Government of KP, [38]	2014	Grey literature
Pakistan Environmental Protection Act, [39]	1997	Grey literature
Government of Punjab, [40]	2001	Grey literature
The Urban Unit-Pakistan, [41]	2023	Grey literature
Institute of Strategic Studies Islamabad, [42]	2023	Grey literature
Ministry of Climate Change of Pakistan, [43]	2023	Grey literature
UN Environment Programme, [44]	2013	Grey literature
International Growth Centre, [10]	2021	Grey literature
Sustainable Development Policy Institute, [45]	2005	Grey literature
Consortium for Development Policy Research, [46]	2023	Grey literature

KP – Khyber Pakhtunkhwa

### Charting and synthesising the data

In accordance with the Arksey and O’Malley framework and the PRISMA-ScR guidelines, we developed a structured, Excel-based charting form to systematically extract descriptive information on policy content, implementation status, and contextual factors. Two reviewers (MZ and HF) independently extracted data from all included scientific and grey literature sources, while a second pair of reviewers (RTB and HM) cross-verified the extracted information through consensus discussions to ensure accuracy and reliability (Appendix S2 in the the **Online Supplementary Document**). The charting process captured key descriptors including the source type, policy name, administrative level (federal or provincial), policy description, implementation details, identified barriers and facilitators, and reported recommendations.

Subsequently, we did a narrative synthesis, guided by the overarching research question. We thematically organised the synthesis around four key domains: policy architecture, existing air quality policies, barriers and facilitators to policy implementation, and recommendations emerging from the literature. We descriptively analysed and corroborated additional contextual information with relevant supporting studies to enhance interpretative depth. This aligns with the aims of a scoping review, which focuses on mapping the scope and characteristics of the available evidence rather than estimating effect sizes or causal relationships.

To enhance transparency, we clarify that barriers and facilitators were synthesised narratively across multiple included sources, while recommendations were either directly extracted from the literature or the authors formulated them based on cross-cutting thematic pattern.

## RESULTS

Results included an overview of the relevant policy architecture (including existing policies in Pakistan), as well as barriers and facilitators to implementation and recommendations for improvement.

### Policy architecture

The national architecture for air quality management has evolved considerably over time [47]. The process began in 1975 with the establishment of the Ministry of Environment, which served as the primary authority for environmental governance. In response to mounting environmental concerns, the Pakistan Environmental Protection Ordinance was enacted in 1983, leading to the formation of the federal Environmental Protection Agency (EPA) in 1987. This was followed by the Pakistan Environmental Protection Act (PEPA) in 1997, which introduced a comprehensive legal framework for environmental protection and enabled the creation of Environmental Protection Agencies at the provincial level. A major shift occurred with the 18th Constitutional Amendment in 2010, which devolved environmental responsibilities to the provinces, granting them the autonomy to develop and implement policies suited to their specific environmental challenges. The passage of the 18th Constitutional Amendment in 2010 fundamentally reshaped the governance of environmental issues in Pakistan by devolving authority over subjects such as environmental protection, pollution control, and ecology from the federal government to the provinces. As a result, the development, implementation, and monitoring of air quality policies now vary considerably across provinces, reflecting differences in institutional capacity, resources, and political priorities. This shift has important implications for our scoping review: post-2010 policy documents are more likely to be issued at the provincial level than at the national level, and coordination challenges between federal and provincial bodies may have contributed to fragmented or uneven policy development. Consequently, our review approach specifically accounted for this governance change to ensure the identification of relevant policy documents across different levels of government [48,49].

### Existing policies on air quality in Pakistan

**Figure 2:**
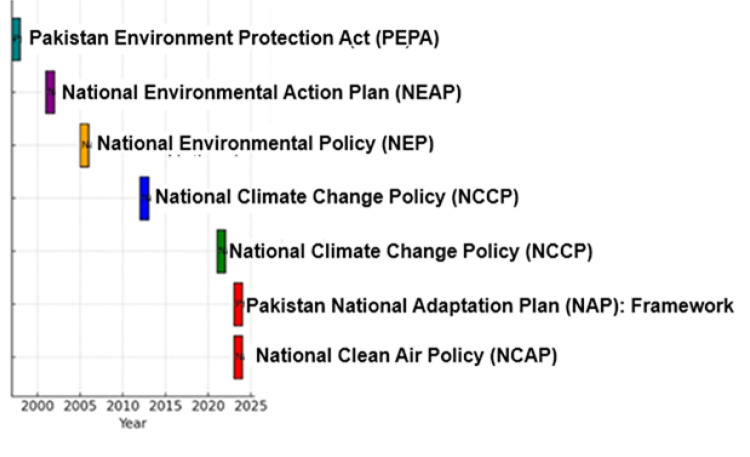
Air quality and climate policy timeline in Pakistan (1997–2023).

This review found seven federal-level policies that directly address air quality. These include the PEPA 1997, the National Environmental Action Plan (NEAP) 2001, the National Environmental Policy (NEP) 2005, the National Climate Change Policy (NCCP) 2012, the NCCP 2021, the Pakistan National Adaptation Plan (NAP) 2023, and the National Clean Air Policy (NCAP) 2023 (**Table 2**). A summary of policies on air quality and climate timeline in Pakistan (**Figure 2**).

**Table 2:** Overview of air quality policies in Pakistan

Policy	Year	Policy description	Implementation
National Clean Air Policy	2023	The first-ever national-level policy identifies a priority intervention in the top five sectors: household, transport, industry, waste and agriculture	No information on implementation yet
Pakistan National Adaptation Plan: Framework	2023	Dealing with climate change through coordination and climate finance mobilisation	No information on implementation yet
National Climate Change Policy	2021	Policy measures for climate resilience and reducing emissions from various sectors; Climate adaptation and mitigation	Key initiatives include the Ten Billion Tree Tsunami Programme and the Clean Green Pakistan Movement [32]; no concrete data on other measures
National Climate Change Policy	2012	Related to the sectors of agriculture, forestry, water, coastal lands, along with their biodiversity and the protection of ecosystems of Pakistan	Development of a framework for implementation of Climate Change Policy
National Environmental Policy	2005	To resolve and conserve the environmental issues through sustainable means, not only to improve the overall development of the state but also to improve the quality of life of its citizens	No information on implementation status
The National Environmental Action Plan	2001	Launched to improve environmental quality, promote sustainable resource use, and ensure that development is environmentally sustainable	Key objectives like the introduction of unleaded gasoline and a reduction of sulphur in diesel have been achieved [50]
Pakistan Environmental Protection Act	1997	Rehabilitation and conservation of the Environment and Sustainable Development Goal	This act established key institutions like the Pakistan Environmental Protection Council and Environment Protection Agencies, which were formed at the provincial level

These policies were developed by public sector institutions, including the Ministry of Climate Change, the Pakistan Environmental Protection Agency, the National Disaster Management Authority, the Ministry of Water Resources, and the Planning Commission of Pakistan, often in collaboration with international organisations. The review also included provincial legislative frameworks that contain actionable components relevant to air quality management. These were formulated by Provincial Environmental Protection Agencies, applicable to four major provinces of Pakistan, including the KP Environmental Protection Act, the Sindh Environmental Protection Act, the Baluchistan Environmental Protection Act, the Punjab Smog Control Policy and the Punjab Clean Air Action Plan (PCAA) (**Table 3**).

**Table 3:** Overview of provincial policies on air quality in Pakistan

Policy	Year	Province	Policy description	Implementation
KP Environment Protection Act	2014	KP	Conservation and rehabilitation of the environment, pollution control, and sustainable development	A Climate Change Cell was established within the Environment Protection Agencies in 2014 to study climate impacts and devise coping strategies. This cell played a pivotal role in formulating the province's first Climate Change Policy in 2017 [51]
Sindh Environment Protection Act	2014	Sindh	Provides a legal framework for the protection, conservation, and improvement of the environment in Sindh province	In 2016, Sindh Environment Protection Agencies published the SEQS, applicable to all industrial and municipal effluents, gaseous emissions, motor vehicle exhaust, noise, and ambient air [52]
Baluchistan Environment Protection Act	2012	Baluchistan	Provides a legal framework for the protection, conservation, and improvement of the environment in Baluchistan province	No implementation because of low institutional capacity, no resources and no political will
Punjab Smog Control Policy	2017	Punjab	Policy suggests health and traffic advisory, traffic management, catalytic converters in vehicles, capacity building in monitoring and forecasting high air pollution episodes	Building upon the 2017 policy, the Punjab government approved the Punjab Clean Air Policy to combat air pollution further
Punjab Clean Air Action Plan	2023	Punjab	Regulation of industrial processes, air pollution, and urban pollution control and development of emergency response systems	No information on implementation yet

KP – Khyber Pakhtunkhwa

### PEPA 1997

PEPA of 1997 is a cornerstone legislation enacted by the National Assembly of Pakistan to safeguard and improve the country's environment. It provides a comprehensive legal framework for managing environmental issues, including environmental impact assessments, hazardous waste management, and the definition and penalisation of environmental offences. PEPA led to the establishment of several key institutional bodies, including the Pakistan Environmental Protection Council, the Pakistan EPA, Provincial Sustainable Development Funds, and Environmental Tribunals. The Council is tasked with coordinating and overseeing NEP, approving the National Environmental Quality Standards (NEQS), and facilitating the implementation of Pakistan’s National Conservation Strategy.

Of relevance to air quality, Sections 11 and 15 of this act prohibit the discharge or emission of pollutants and noise beyond the NEQS. For air quality, NEQS sets limits on pollutants like PM (PM10 and PM2.5), SO2, nitrogen oxides, CO, and ozone, aimed at reducing respiratory issues and global warming. However, the NEQS for PM2.5 and PM10 are not aligned with the latest WHO air quality guidelines [48]. Implementation and enforcement of these standards remain a challenge, as actual pollution levels in major cities often exceed even national limits. Section 16 empowers the federal government to enforce the installation of pollution control devices, the use of specific fuels, and the maintenance of motor vehicles to manage air and noise pollution. NEQS set fuel-related limits, including the sulphur content of fuel oil (1%), emission standards for power plants, and vehicular emissions, but enforcement remains a challenge. The act also imposes penalties for non-compliance with these national standards, including significant fines, imprisonment, and potential facility closures [49]. Overall, while the PEPA provides a comprehensive framework for environmental protection, its success has been limited by enforcement challenges, resource limitations, and varying levels of compliance across different sectors.

### NEAP 2001

The Pakistan Environmental Protection Council approved the NEAP in February 2001 as an umbrella programme to address environmental concerns holistically. The Pakistan EPA was primarily responsible for implementing this policy, with oversight from the Ministry of Environment. The NEAP aimed to improve air quality through several key provisions that mitigate air pollution. The plan advocated for the introduction of unleaded gasoline to reduce lead emissions from vehicles, a significant source of urban air pollution; recommended lowering sulphur levels in diesel fuel to decrease SO2 emissions (which contribute to smog and respiratory problems); and emphasised the adoption of cleaner technologies in industries and transportation to minimise emissions of PM and other pollutants. The plan also proposed initiatives to raise public awareness about the health impacts of air pollution, encourage community participation in air quality management and improve the capabilities of environmental protection agencies at the federal and provincial levels to monitor and enforce air quality standards effectively. While the NEAP aims to address environmental degradation and promote sustainable development, information on its implementation progress and effectiveness is limited, indicating challenges in achieving its objectives.

### NEP 2005

The NEP was approved by the Federal Cabinet in 2005. The Ministry of Environment was primarily responsible for its implementation, coordinating with various federal and provincial agencies. This policy provided an overarching framework for institutional strengthening and governance for addressing a range of environmental issues in Pakistan, including pollution control. Provincial governments were advised to devise their own strategies, plans, and programmes in pursuit of this policy, with the level of priority and commitment varying among provinces. While Sindh was one of the first to enact such a policy at the provincial level, other provinces, including Punjab, KP, and Baluchistan, also followed suit with their own environmental policies and laws in subsequent years, each adapting the broader framework provided by national environmental laws to meet regional needs [20]. The policy is implemented through various measures, including development planning, legislation, capacity development, economic instruments, education, and public-private partnerships. A committee is responsible for monitoring the policy's progress and reporting on its implementation every six months. The establishment and enforcement of indoor and outdoor air quality standards were core objectives of the ten-year NEP (2005–15), which has not yet been implemented.

### NCCP 2013

This policy was developed and implemented in 2013, and recently updated in 2021, reflecting a more ambitious and comprehensive approach to climate action, incorporating the latest scientific developments and integrating climate resilience and mitigation strategies. Regarding air quality, the updated NCCP identifies the reduction of air pollution as a co-benefit of mitigating greenhouse gas emissions and outlines measures to enhance air quality. These measures include promoting cleaner production technologies, enforcing environmental regulations, and developing urban planning strategies that reduce vehicular emissions. However, the NCCP's broad scope, encompassing 24 objectives and 215 actions, lacks prioritisation and clear accountability mechanisms. This complexity complicates the implementation process and may dilute focus on critical areas [53].

### NAP 2023

The Federal Cabinet approved NAP 2023 in July 2023. The Ministry of Climate Change and Environmental Coordination is responsible for implementing this policy. This plan recommends developing and implementing clean-air investment plans for key sectors, including domestic cooking, transport, industry, agriculture, and waste burning, to meet Pakistani air quality standards. It also emphasises the need to establish and enforce air quality standards in these sectors. Additionally, the NAP suggests conducting air governance assessments and implementing improvement plans to strengthen air quality management. As of now, the NAP has been officially launched, but detailed information on its implementation status is limited [50].

### NCAP 2023

Approved by the Federal Cabinet of Pakistan in March 2023, the NCAP is being implemented by the Ministry of Climate Change, with support from provincial EPAs, which are responsible for developing and executing provincial clean air action plans (CAAPs). As a nationwide initiative, the policy aims to combat air pollution through a range of targeted interventions, including the enhancement of air quality monitoring and data collection, reduction of vehicular emissions, promotion of clean energy, control of industrial emissions, improved waste management practices (including the prevention of open burning), public awareness and education campaigns, health and environmental impact assessments, and coordination of provincial and local-level strategies, alongside international cooperation. The policy underscores the significant economic, social, and health burdens of air pollution and aligns its strategy with existing legal frameworks and environmental policies. It calls for multi-sectoral interventions across the transport, industrial, agricultural, residential, and municipal sectors, identifying priority actions to catalyse coordinated efforts. Notable interventions include implementing Euro-5 and Euro-6 fuel quality standards, enforcing industrial emission standards, preventing agricultural residue burning, eliminating open municipal waste burning, and promoting low-emission cooking technologies. While the policy has been formally approved and provincial action plans are currently in development, comprehensive information on its implementation progress remains limited at this stage.

### Punjab Smog Controlling Policy 2017

This policy was adopted by the Environment Protection Department, Government of Punjab, and it includes issuing health and travel advisories during smog conditions, shutting down major smoke-emitting industries, installing catalytic converters in vehicles, and improving traffic management. It also suggests creating woodlands around cities, designing road shoulders to control dust, greening industrial processes, and building capacity to monitor air pollution. Additionally, it recommends regional environmental agreements between India and Pakistan.

### PCAA 2023

This plan was devised by the Environment Protection Department, Government of Punjab, and encompasses six main sectors: industry, transport, agriculture, environment, local government, and the housing and urban planning sector, each with specific policy measures. The Punjab Environment Protection Department is primarily responsible for implementing this plan, with support from various provincial departments, including Transport, Agriculture, Industries, Local Government, Housing, Urban Development, Energy, and Labour. It promotes technology diffusion, capacity building, and industry relocation for industrial processes. To combat air and vehicle pollution, it advocates for low-emission vehicles, catalytic converters, low-sulphur fuels, and mandatory bus transport for students. Urban pollution control focuses on increasing tree cover, promoting non-motorised travel, and controlling road dust. Alternatives to crop residue burning include restrictions, farmer awareness, and the use of biomass for energy. The plan also enhances monitoring, alerting, and emergency response systems and strengthens the legal framework and organisational capacity by introducing the PCAA, which sets emission reduction and monitoring requirements and builds capacity and human resources. The PCAA represents a significant effort by the provincial government to address air pollution. While substantial financial resources have been allocated, ongoing monitoring and transparent reporting are essential to ensure the plan's success and to make necessary adjustments based on its performance.

The provinces of KP, Balochistan, and Sindh have developed their own CAAPs to address air quality issues. These plans were formulated with support from national agencies and international organisations to ensure a coordinated approach to air pollution mitigation. These provincial plans are part of Pakistan's broader efforts to improve air quality and align with national and international standards. However, challenges remain in monitoring, enforcement, and achieving the desired air quality improvements.

### Other provincial policies on air quality in Pakistan

KP developed a Climate Change Action Plan, including measures to address air pollution, which was implemented by the provincial Environmental Protection Agency. Balochistan has also created a CAAP with the support of national and international stakeholders. The Environmental Protection Agency of Balochistan oversees the implementation of the CAAP. Similarly, Sindh has developed a CAAP, with assessments conducted to identify pollution sources and potential reduction measures. The Environment, Climate Change, and Coastal Development Department of Sindh is responsible for implementing the CAAP.

### Barriers to the implementation of air quality policies in Pakistan

#### General public

Low literacy levels, limited engagement, and the absence of reliable information sources leave much of the population unaware of the health risks posed by air pollution, dampening public demand for cleaner air and, in turn, the pressure on policymakers to act [32,51]. Household reliance on low-quality, highly polluting fuels – driven by pricing distortions, rationing, and scarce cleaner-energy options – further degrades ambient air quality [53]. Compounding these outdoor sources, indoor exposures receive little attention: routine use of solid fuels for cooking and widespread environmental tobacco smoke mean that indoor air pollution remains a substantial yet under-recognised contributor to the overall burden [49].

#### Government and policymakers

In Pakistan, air-quality management suffers from low political prioritisation: policymakers and local authorities seldom treat pollution control as urgent, resulting in weak legislative focus and sluggish action [32,51]. Even where environmental standards exist, lax monitoring and enforcement undermine their impact, as no robust system provides regular surveillance or feedback to refine regulations [20,48,52,53]. These shortcomings are compounded by wide research and data gaps – baseline pollution levels, indoor exposures, fuel-use patterns, and long-term health risks remain poorly characterised, and little work addresses practical issues such as kitchen design or behaviour change – leaving decision-makers without the evidence needed for effective, targeted interventions [20,48,49,53].

#### Industries and the energy sector

Many industrial stakeholders remain either unaware of – or unconvinced by – the environmental costs of their operations, giving them little incentive to adopt cleaner technologies and delaying emission cuts [32,51]. This inertia is compounded by Pakistan’s continued dependence on low-grade fuels, recently reinforced by moves to prioritise Thar coal, while policy support for cleaner energy remains scant [48]. Inefficient energy use in commerce and an ageing, diesel-subsidised vehicle fleet further aggravate urban air quality, binding the country to a high-pollution trajectory [48].

#### Environmental and structural challenges

Pakistan’s limited indigenous energy reserves, coupled with widespread reliance on outdated, inefficient technologies, drive high energy intensity and elevated pollution levels. Fuel-rationing policies and pricing distortions exacerbate the problem by keeping low-quality fuels cheap and readily available, while cleaner alternatives remain economically out of reach, locking the country into a cycle of dirty-energy dependence [48].

#### Facilitators for air quality policy implementation in Pakistan

Published literature highlights a few key facilitators for the effective implementation of air quality policies in Pakistan: effective air-quality governance in Pakistan hinges on integrating climate-change considerations into policies at every tier – national, provincial, divisional, and local – backed by strong institutional arrangements that ensure coordinated implementation [54]. Such integration should be coupled with cross-sectoral action plans that align environment, health, energy, transport, and industry agencies around clear mitigation targets [20]. Embedding sustainability in national development agendas fosters eco-friendly attitudes and raises public awareness of environmental degradation, while government promotion of cleaner industrial technologies – advanced combustion processes and energy-efficiency measures – demonstrates how technological progress can reinforce policy goals and improve air quality [20,54].

#### Recommendations for improved policy implementation

A robust air-quality strategy in Pakistan must begin with stronger governance: installing formal feedback loops to identify policy gaps, fostering collaboration among industry, the public and regulators, and giving universities a leading role in research-driven innovation can counteract the chronic deficit of political will [32,51]. Complementing this, legislation should pivot from command-and-control rules to market-based incentives; financial rewards and penalties, integrated climate-energy-air policy, and an outright ban on open crop burning – especially during smog season – would align economic motives with pollution reduction [20,32,48,49,52].

Reliable data are equally pivotal. Deploying low-cost continuous sensors, coupling satellite imagery with ground stations, and experimenting with wireless-sensor networks or e-drones would close critical monitoring gaps and extend the Air Quality Health Index beyond major cities [48,49,53]. These technological advances must feed into transparent public reporting to keep regulators, industries and citizens openly accountable.

Public engagement then sustains momentum. Early environmental education, consumer incentives for clean production, and industry self-monitoring systems can normalise pollution accountability, while financial support and demonstration projects can help farmers replace crop burning with composting, biogas or in-field residue management [20,47-49].

Finally, structural investments will lock in long-term gains: expanding urban tree canopies to absorb pollutants and temper heat islands; scaling waste-to-energy technologies – controlled incineration, anaerobic digestion, biodiesel from waste oils – to cut landfill methane and diesel use; enforcing fuel-desulphurisation and other clean-production upgrades across refineries and factories to curb sulphur-linked emissions; and incentivising viable alternatives to stubble burning [48-50,53]. Together, these coordinated measures create a clear, evidence-based roadmap for cleaner air and healthier communities.

## DISCUSSION

Pakistan now ranks third among the world’s most polluted countries [52]. Although most government documents frame the problem as one of environmental sustainability, it is necessary to reframe it as an acute public-health crisis demanding swift, coordinated action [48]. We found wide gaps between policy intent and real-world implementation. Despite a growing portfolio of regulations, weak enforcement, fragmented data systems and shifting political priorities blunt their impact.

Inter-governmental fragmentation within a federal structure is a central obstacle. Since the 18th Constitutional Amendment devolved many environmental powers, standards now vary by province and federal-provincial coordination has deteriorated. Statutory bodies such as the Environmental Protection Council meet infrequently, limiting oversight, while provincial regulators lack the resources to enforce even basic vehicle-emission or Euro V fuel standards [9,10]. Industrial emissions remain a significant problem, worsened by the use of small diesel generators, outdated technology, and lax regulations [10]. Our findings echo the report Charting Pakistan’s Air-Quality Policy Landscape, which identified poor inter-agency coordination, inadequate funding and the absence of a centralised data repository as key bottlenecks [9].

Transport and industry remain large, under-regulated emitters. Policies such as the PCAA call for low-emission vehicles and industrial technology upgrades, yet uptake is slow owing to resource constraints and limited public awareness. Rural reliance on solid fuels for cooking and heating further elevates ambient and household pollution, while the roll-out of cleaner energy alternatives remains patchy. Limited public engagement and inefficient pollution-level tracking result from the absence of, or inadequate funding for, extensive air quality monitoring networks and public awareness initiatives. Outdated infrastructure, an unreliable electricity grid, inflationary pressure and recurrent allegations of petty corruption erode programme credibility and continuity [11,54,55].

Effective management is not possible without reliable data. Air-quality monitoring networks are sparse, under-funded and concentrated in major cities; real-time public reporting is inconsistent. To enhance environmental policy implementation, it is essential to measure the effectiveness of existing initiatives, such as the Pakistan Clean Air Program and Nationally Determined Contributions. Creating an integrated database with projections for emissions, population growth, and socio-economic factors can serve as a foundation for cost-effective emission control strategies. Relevant government agencies must quantify the impact of air quality programmes to ensure policies address both greenhouse gas emissions and short-lived climate pollutants. Provinces should develop localised Clean Air Plans with specific priorities that reflect regional challenges and stakeholder involvement [24,56]. Citizen-science initiatives such as the Pakistan Air Quality Initiative demonstrate how partnerships between government, academia and civil society can fill data gaps, improve transparency and rebuild public trust [11,54]. Formalising such collaborations would also generate the emissions evidence needed to move from command-and-control rules to market-based instruments – emissions trading, pollution taxes or fuel-sulphur levies – that have proven cost-effective elsewhere [25,26,28].

There is a need to transition from command-and-control mechanisms to market incentives – tax breaks, subsidies, or pollution levies – that reward cleaner fuels, advanced technologies, and sustainable farming practices, such as alternatives to open stubble burning. This shift could lead to cost-effective pollution abatement, especially in sectors where regulatory compliance remains challenging. Implementing these strategies, however, requires robust data on emissions and compliance, underscoring the urgent need to enhance monitoring capacities and data infrastructure in Pakistan [57–59]. Addressing Pakistan’s air quality challenges requires a multi-pronged approach involving better policy coordination, cost-effective technologies, enhanced public awareness, and the inclusion of informal regulators. A coherent reform agenda would begin by creating a federal-provincial task force with a legal mandate to harmonise standards, share data and hold all jurisdictions to time-bound pollution-reduction targets. Quantifying the economic and social costs of pollution and prioritising policies based on their broader benefits will help the country achieve its emission reduction targets. Public momentum can be built by weaving air-quality content into school curricula, financing media campaigns and scaling community monitoring so that public concern translates into political pressure. Finally, sustained investment in renewable power and modern household energy will accelerate Pakistan’s shift away from high-carbon, polluting fuels, simultaneously curbing greenhouse gases and short-lived climate pollutants. Dialogue among affected communities, decision-makers, researchers, and policymakers is critical to align these technical priorities with political realities.

This study has some limitations that should be acknowledged. Unlike conventional scoping reviews that explore broader aspects of a topic, we focused on identifying existing policy documents on air quality in Pakistan to align with the aims of a larger stakeholder-engaged project. The key limitation is that we relied on peer-reviewed literature; we obtained policy reports, government documents, and other grey sources only from websites. We assessed implementation status only from published literature and publicly available reports, which may limit completeness. This was an *a priori* decision, which means essential insights on implementation practice may be under-represented. In addition, the absence of a standardised search strategy for policy documents, the restriction to English-language sources, potential publication and policy-availability bias, and the lack of formal quality assessment of included documents may have further limited the scope and rigour of our findings.

## CONCLUSIONS

Addressing Pakistan’s air quality issues requires a holistic approach that includes strengthening inter-agency coordination, adopting advanced pollution control technologies, engaging informal regulatory bodies, and moving towards incentive-based regulatory mechanisms. Increasing public awareness and integrating environmental concerns into economic planning will also be crucial for sustainable improvements in air quality.

## Additional material


Online Supplementary Document
