# Supplementary material for: Current landscape and future directions of policies addressing air quality improvement in Pakistan: a scoping review
Source: J Glob Health. 2025 Dec 29;15:04349. doi: 10.7189/jogh.15.04349 (PMC12746533; doi:10.7189/jogh.15.04349)
Supplement: Online Supplementary Document [file jogh-15-04349-s001.pdf]

Supplement to: Zahid M, Baig RT, Fatima H, Mahmood H, Shakeel P, Khan A, Fernandes G, Soofi S, Yusuf OM, Ariff S, Bauld L; RESPIRE collaboration. Current landscape and future directions of policies addressing air quality improvement in Pakistan: a scoping review. J Glob Health. 2025;15:04349.

SUPPLEMENTARY MATERIAL

Table of contents

1. **Search strategies and search hits……………………………………………………………………1**
2. **Consensus data extraction sheet……………………………………………………………………4**

Appendix S1: SEARCH STRATEGIES AND SEARCH HITS

Search strings:

- **Concept 1 / String 1**
  Air Pollution OR air quality OR Ambient Air OR Particulate Matter OR PM 2.5 OR Air Pollutants OR Vehicle Emission OR Traffic Pollution OR Indoor Air Quality OR Outdoor Air Pollution OR Fog OR Smoke OR Smog
- **Concept 2 / String 2**
  Regulation OR Air Quality Regulation OR Policy OR Air Pollution Policy OR National Clean Air Policy OR Environmental Policy OR Ministry of Environment OR Climate Change OR Government OR laws OR Strategy OR Guideline OR Governance OR Framework OR Action OR Action Plan OR Program
- **Concept 3 / String 3**
  Pakistan OR Lahore OR Karachi OR Islamabad OR Faisalabad OR Peshawar OR Rawalpindi

SEARCH hits: Pubmed

Date: 17th May 2024

Table 1:

SNO.	SEARCH TERMS	RESULTS
1	**Concept 1**<br><br>Air Pollution"[Mesh] OR "air quality*" OR "Ambient Air" OR "Particulate Matter"[Mesh] OR PM 2.5 OR "Air Pollutants"[Mesh] OR "Vehicle Emission*" OR "Traffic Pollution" OR "Indoor Air Quality"[Mesh] OR "Outdoor Air Pollution"[Mesh] OR Fog OR Smoke OR Smog	145,128
2	**Concept 2**<br><br>Regulation* OR "Air Quality Regulation"[Mesh] OR Policy OR "Air Pollution Policy*"[Mesh] OR "National Clean Air Policy"[Mesh] OR "Environmental Policy*"[Mesh] OR "Ministery of Environment"[Mesh] OR "Climate Change"[Mesh] OR Government[Mesh] OR laws OR Strategy OR Guideline* OR Governance OR Framework OR Action OR Action Plan OR Program	2,636,156
3	**Concept 3**<br><br>Pakistan* OR Lahore OR Karachi OR Islamabad OR Faisalabad OR Peshawar OR Rawalpindi	29,092
4	**Concept 1 AND Concept 2 AND Concept 3**<br><br>("Air Pollution"[Mesh] OR "air quality*" OR "Ambient Air" OR "Particulate Matter"[Mesh] OR PM 2.5 OR "Air Pollutants"[Mesh] OR "Vehicle Emission*" OR "Traffic Pollution" OR "Indoor Air Quality"[Mesh] OR "Outdoor Air Pollution"[Mesh] OR Fog OR Smoke OR Smog) AND (Regulation* OR "Air Quality Regulation"[Mesh] OR Policy OR "Air Pollution Policy*"[Mesh] OR "National Clean Air Policy"[Mesh] OR "Environmental Policy*"[Mesh] OR "Ministery of Environment"[Mesh] OR "Climate Change"[Mesh] OR Government[Mesh] OR laws OR Strategy OR Guideline* OR Governance OR Framework OR Action OR Action Plan OR Program) AND (Pakistan* OR Lahore OR Karachi OR Islamabad OR Faislabad OR Peshawar OR Rawalpindi)	936

SEARCH hits: Scopus

17th May 2024

SNO.	SEARCH TERMS	RESULTS
1	**Concept 1**<br><br>Air Pollution"[Mesh] OR "air quality*" OR "Ambient Air" OR "Particulate Matter"[Mesh] OR PM 2.5 OR "Air Pollutants"[Mesh] OR "Vehicle Emission*" OR "Traffic Pollution" OR "Indoor Air Quality"[Mesh] OR "Outdoor Air Pollution"[Mesh] OR Fog OR Smoke OR Smog	9,439
2	**Concept 2**<br><br>Regulation* OR "Air Quality Regulation"[Mesh] OR Policy OR "Air Pollution Policy*"[Mesh] OR "National Clean Air Policy"[Mesh] OR "Environmental Policy*"[Mesh] OR "Ministery of Environment"[Mesh] OR "Climate Change"[Mesh] OR Government[Mesh] OR laws OR Strategy OR Guideline* OR Governance OR Framework OR Action OR Action Plan OR Program	1,196
3	**Concept 3**<br><br>Pakistan* OR Lahore OR Karachi OR Islamabad OR Faisalabad OR Peshawar OR Rawalpindi	110,343
4	**Concept 1 AND Concept 2 AND Concept 3**<br><br>("Air Pollution"[Mesh] OR "air quality*" OR "Ambient Air" OR "Particulate Matter"[Mesh] OR PM 2.5 OR "Air Pollutants"[Mesh] OR "Vehicle Emission*" OR "Traffic Pollution" OR "Indoor Air Quality"[Mesh] OR "Outdoor Air Pollution"[Mesh] OR Fog OR Smoke OR Smog) AND (Regulation* OR "Air Quality Regulation"[Mesh] OR Policy OR "Air Pollution Policy*"[Mesh] OR "National Clean Air Policy"[Mesh] OR "Environmental Policy*"[Mesh] OR "Ministery of Environment"[Mesh] OR "Climate Change"[Mesh] OR Government[Mesh] OR laws OR Strategy OR Guideline* OR Governance OR Framework OR Action OR Action Plan OR Program) AND (Pakistan* OR Lahore OR Karachi OR Islamabad OR Faislabad OR Peshawar OR Rawalpindi)	164

Appendix S2: Consensus Data Extraction Sheet

ID	Aziz 2006	Hussain 2019	Mir 2024	Mir 2016	Anwar 2021	Anjum 2021	Colbeck 2010	Ahmed 2023
**Title**	Towards establishing air quality guidelines for Pakistan.	Divisional disparities on climate change adaptation and mitigation in Punjab, Pakistan: local perceptions, vulnerabilities, and policy implications.	Black carbon emissions inventory and scenario analysis for Pakistan.	Analysis of baseline and alternative air quality scenarios for Pakistan: an integrated approach.	Emerging challenges of air pollution and particulate matter in China, India, and Pakistan and mitigating solutions.	An Emerged Challenge of Air Pollution and Ever-Increasing Particulate Matter in Pakistan; A Critical Review.	The state of indoor air quality in Pakistan--a review.	Climate change adaption strategies in urban communities: new evidence from Islamabad, Pakistan.
**Policy or Program**	The protection of ambient air quality in Pakistan is provided through the enforcement of the National Environmental Quality Standards, introduced in July 1996. These standards impose limits on the concentration of	-	Nationally Determined Contributions (NDCs)<br><br>The ˜National Clean Air Policy (NCAP)" is the country's first comprehensive framework for enhancing air quality. It identifies priority interventions in transportation,	Emission control policies, measures, and regulatory framework: Pakistan Clean Air Program (PCAP): In 2005, the Government of Pakistan elaborated the PCAP, which contains a list of interventions for improving air quality. The	(Pakistan only) 1) Pakistan Clean Air Program: February 2001. Short-term measures: establishing environmental squads in all major cities, restrictions on converting vehicles from gasoline to second-hand diesel engines,	Factories Act 1934	Standards for ambient and indoor air quality (National Environment Policy of Pakistan 2005).<br><br>Prohibition of Smoking and Protection of Non Smokers Health Ordinance: In 2002, Pakistan introduced the	-

ID	Aziz 2006	Hussain 2019	Mir 2024	Mir 2016	Anwar 2021	Anjum 2021	Colbeck 2010	Ahmed 2023
	specified gaseous emissions from industrial units and automobiles.		industry, agriculture, waste, and residential sectors, and recommends five key mitigation actions for each sector to combat air pollution. National Environment Action Plan (NEAP) 2001: Identified clean air as a core focus (Pak-EPA, 2003). Subsequently, the "Pakistan Clean Air Program (PCAP)" was introduced in 2005, involving short and long-term actions to be taken collectively by the government and relevant agencies. The PCAP targeted major sources of air pollution, including	PCAP includes interventions bearing on (a) vehicular emissions, (b) industrial emissions, (c) burning of solid waste, and (d) natural dust. PCAP's main objective is to reduce the health and economic impacts of air pollution by implementing a number of short-term and long-term measures that require action at all levels of government. National Energy Conservation Policy (2006). National Renewable Energy Policy (2006). Policy for Development of Renewable Energy for Power	traffic management, and token tax for diesel vehicles. Long-term measures: public education and awareness, review of regulations, laws and policies, and afforestation.<br><br>2) National Climate Change Policy: September 2012. Consideration of new fuel-efficient aircrafts to curb carbon emission, improving air traffic management, free flight routes instead of specified flight routes, and improved weather services. Promoting CNG		Prohibition of Smoking and Protection of Non Smokers Health Ordinance which instigated a ban on smoking in closed places, health facilities, educational facilities, and on public transportation. However, the legislation is not implemented, and in 2008, the Government issued guidelines for the creation of designated smoking areas.	

ID	Aziz 2006	Hussain 2019	Mir 2024	Mir 2016	Anwar 2021	Anjum 2021	Colbeck 2010	Ahmed 2023
			emissions from road transport, industries, solid waste combustion, and natural dust or transboundary pollution. To establish air quality guidelines, the ˜National Environment Quality Standards (NEQS)" were developed between 2009 and 2010, covering ambient air and motor vehicle exhaust and addressing key pollutants. The provincial Environmental Protection Departments (EPDs) played a crucial role in engaging local governments to enforce environmental regulations outlined in	Generation (2006). National Forest Policy (2010). The National Environment Policy (NEP) 2005: It calls for setting of regulations for ambient and indoor air quality, standards for vehicles, industrial emissions and fuel quality, energy conservation, and building codes. The framework for air quality management system in the country dates back to 1993, when the National Environmental Quality Standards (NEQS) were developed under the 1983 Environmental	in the transportation sector and adopting environmentally friendly technologies.<br><br>3) Adopting Euro II standards for vehicular emissions (Ref: Smog Control Policy): 21/10/2017. Adoption of Euro II standards followed by strict inspection of vehicular emissions. Extending low-sulfur fuel to petrol vehicles. Restricting the import of second-hand vehicles which do not comply with the Euro II standard.<br><br>4)Controlling burning of crop residues and			

ID	Aziz 2006	Hussain 2019	Mir 2024	Mir 2016	Anwar 2021	Anjum 2021	Colbeck 2010	Ahmed 2023
			˜Pakistan Environment Protection Act (PEPA)" of 1997, which forms the cornerstone of environmental legislation in the country.<br><br>Punjab province: Policy on Controlling Smog, the Punjab Clean Air Action Plan, and Punjab Clean Air Policy (with phased action plan) 2023. These policies involve comprehensive action plans spanning multiple sectors and incorporate clear mitigation measures with the aim of reducing air pollution.<br><br>Pakistan's Ministry of Climate Change	Protection Ordinance. In December 1999, the Pakistan Environmental Protection Council (PEPC) approved a revised version of the NEQS, and they became effective in August 2000. In 2010, Pak-EPA drafted NEQS for ambient air that cover several major pollutants: (a) SO2, (b) NOx, (c) O3, (d) suspended particulate matter (SPM), (e) PM2.5, (f) Pb, and (g) CO. PEPC approved NEQS standards in March 2010, and the official notifications in the Gazette of Pakistan were made on November 26, 2010. Under the	Municipal waste (Ref: Smog Control Policy): 21/10/2017 Preparation and execution of plans for crop residue safe disposal. Insurance of 75% efficiency of solid waste collection. No approval of housing societies unless a solid waste management system is provided.<br><br>5) Greening Industrial Processes (Ref: Smog Control Policy): 21/10/2017 Introduction of efficient technologies in industries, specifically in thermal power plants, steel mills, and cement industry.			

ID	Aziz 2006	Hussain 2019	Mir 2024	Mir 2016	Anwar 2021	Anjum 2021	Colbeck 2010	Ahmed 2023
			and Environmental Coordination has implemented measures aimed at reducing emissions from new brick kilns that are still employing outdated technology. These efforts have focused on promoting the use of relatively more environmentally friendly zigzag kilns.<br><br>Ongoing efforts aim to promote practices among farmers that encourage plowing under a larger portion of crop residues. Such practices not only enhance soil fertility and organic content but also have the potential to	NEQS for motor vehicle exhaust and noise, the environment ministry has set standards (maximum permissible limits and measuring methods) and measuring methods for smoke, carbon monoxide, and noise. Recently, Euro-II standards for vehicle emissions were adopted for newly registered vehicles. Pakistan signed the United Nations Framework Convention on Climate Change (UNFCCC) as a non-Annex I Party in June 1994. The country, subsequently, adopted the Kyoto Protocol	6) Planned development of Industrial and Urban units (Ref: Smog Control Policy): 21/10/2017 Developing a master plan for land use for cities and adjacent countrysides.<br><br>7) Prohibition of certain emissions or discharge (Section 11) (Ref: Pakistan Environmental Protection Act): 1997 Subsection (1): no person shall emit air pollutants in concentrations exceeding the National Environment Quality Standards (NEQS). Subsection (2): the federal government is			

ID	Aziz 2006	Hussain 2019	Mir 2024	Mir 2016	Anwar 2021	Anjum 2021	Colbeck 2010	Ahmed 2023
			significantly reduce the proportion of residues burned in the fields.<br><br>TABLES S1 and S2 in Supplementary material.	in 1997 and acceded to it on 11 January 2005. The government recently approved the draft of National Policy on Climate Change, which provides a framework for addressing the issues that Pakistan face or will face in future due to the changing climate.	responsible for levying pollution charges on parties who fail to comply with the provisions mentioned above.			
**Barriers**	The National Environmental Quality Standard were imposed without giving careful consideration to the desired ambient air quality. As such, the real objective of minimizing the risk to health of humans and animals and of damage to the	Acute shortage of knowledge and awareness regarding measures of mitigation and synchronization (adaptation). Lack of interest on severity of the environmental issue at not only individual and divisional level but at governmental	Estimating BC emissions accurately in Pakistan's industrial sector poses significant challenges due to the diverse range of fuels, combustors, and control devices employed in this sector. Absence of specific policies targeting BC emissions. Lack	-	-	Challenges of Air Pollution Management in Pakistan:<br><br>1) Lack of awareness: among stakeholders, reluctance to change, absence of reliable information sources.<br>2) Political will: Throughout the history of	There is very little published literature available regarding indoor air pollution in Pakistan. Lack of baseline assessment of pollution and exposure, fuel use, and house structure. Little research has been directed towards kitchen design and	There is usually no legal framework or institutional determination to implement national policies. There is a dearth of academic inquiry into urban concerns generally and in the context of cities and climate change specifically.

ID	Aziz 2006	Hussain 2019	Mir 2024	Mir 2016	Anwar 2021	Anjum 2021	Colbeck 2010	Ahmed 2023
	environment in general may not be being achieved. There is therefore a need to establish national air quality guidelines in Pakistan. No reliable epidemiological/ toxicological studies have been undertaken in Pakistan to provide recommended pollutant concentration limits in order to protect human health. Regular monitoring of ambient air quality is still not systematic in Pakistan. All the available information is based on random and short-term sampling conducted to assess the	level as well, because government has not taken any specific steps towards dispersing education, and raising awareness among people about the seriousness of climate change impacts. The overall country's economic planning has not included a serious issue such as climate change in the policy formulation agenda and is continuously ignored in budgetary meetings.	of continuous monitoring and enforcement necessary to ensure successful implementation.			Pakistan, key stakeholders have ignored meaningful policy solutions like the development and enforcement of air quality standards for indoor and outdoor environments, a major goal of the 10-year national environmental policy (2005-2015). Failure of the 2002 Prohibition of Smoking and Protection of Non-Smokers Health Ordinance, which despite being a part of the legislation, was not properly enforced, and in 2008, the Government abandoned it and issued guidelines for the creation of	behavior change although these could offer significant improvements. There is a scarcity on studies on the levels of indoor pollution due to environmental tobacco smoke (ETS) in Pakistan. As smoking in indoor environments is very common, ETS makes a significant contribution to indoor air pollution in the country. Indoor air pollution has received little attention in Pakistan because of lack of awareness among the population and policy makers regarding the association of indoor air	

ID	Aziz 2006	Hussain 2019	Mir 2024	Mir 2016	Anwar 2021	Anjum 2021	Colbeck 2010	Ahmed 2023
	concentrations of various pollutants.					designated smoking areas. 3) Lack of proper health, industrial and monitoring policies and implementation: There is no policy feedback mechanism at regulatory authorities in Pakistan. Pak-NEQS have been set and revised on some occasions, but there is no such feedback on whether the policies were implemented in true spirit or not. Lack of support by the government and lack of awareness among the public and legislative members in Pakistan. 4) Use of improper fuel: Historically	pollution and ill health.	

ID	Aziz 2006	Hussain 2019	Mir 2024	Mir 2016	Anwar 2021	Anjum 2021	Colbeck 2010	Ahmed 2023
						Pakistan has used highly polluting and poor-quality fuels because of fuel rationing, price distortions, and an overall unconcern with the generated air pollution. The country's choice of moving away from oil imports and focusing on the use of Thar coal will have a worsening impact on the state of air quality in the coming years. 5) Increasing energy demands: Pakistan does not have indigenous reserves to meet its energy demands and uses energy in highly inefficient ways at the commercial		

ID	Aziz 2006	Hussain 2019	Mir 2024	Mir 2016	Anwar 2021	Anjum 2021	Colbeck 2010	Ahmed 2023
						scale. Many factors are increasing the energy intensity of Pakistan. Some of them include the use of aged vehicles and the price subsidies on diesel and thermal power generation.		
Facilitators	-	In order to integrate climate change into national agenda, policymakers not only need to include climate change as a national issue while making policies at every level including national, provincial, divisional, and local level, but also need to make such institutional arrangements, through which implementation of these policies is ensured in an	These policies involve comprehensive action plans spanning multiple sectors and incorporate clear mitigation measures with the aim of reducing air pollution in the province. Pakistan government has taken steps to encourage cleaner and more efficient industrial practices. This includes promoting the adoption of	-	-	-	-	-

ID	Aziz 2006	Hussain 2019	Mir 2024	Mir 2016	Anwar 2021	Anjum 2021	Colbeck 2010	Ahmed 2023
		effective and efficient way. The purpose of incorporating climate change into national development plan should be to address and promote sustainable activities, build eco-friendly attitudes, and spread knowledge about environmental degradation.	cleaner technologies, enhancing combustion processes, and advocating for energy efficiency.					

ID	Aziz 2006	Hussain 2019	Mir 2024	Mir 2016	Anwar 2021	Anjum 2021	Colbeck 2010	Ahmed 2023
**Recommendations**	Proposed air quality guidelines for Pakistan:<br><br>1) Carbon dioxide: WHO provides guidelines for 1-hour and 8-hour averaging times as 30 000 Î¼g/m and 10 000 Î¼g/m respectively. Most countries in the Regions have employed similar standards and the same levels for CO are therefore proposed for Pakistan.<br>2) Ozone: Keeping in view the averaging times adopted in Indonesia, Sri Lanka and Thailand and the prevailing local conditions, the suggested O level for Pakistan over 1-	Governmental policies should be updated to create awareness among masses and reduce the effects of climate change. This can be done by creating training manuals specifically developed to cater the needs of diverse demographics according to their knowledge levels, regions, and sectors. These training manuals should be established to focus on altering behaviors of the population through increasing knowledge about climate change impacts formed by specific demographics, procedures, and actions required	Prohibiting open burning of municipal solid waste and crop residue. Prioritizing efforts to replace inefficient and polluting small coal boilers with cleaner alternatives such as natural gas and electricity. Adopting filter-based standards, such as Euro VI emission standards, for heavy-duty diesel trucks and buses can lead to significant BC emission reductions of over 95%, including other ultrafine particulates. Full implementation of emission standards for industrial boilers would also result in substantial BC	Applying advanced emission control technology to all large sources in Pakistan.	Potential solutions and the way forward:<br><br>1) Improving legislations: Policy measures based on economic incentives and market-based instruments for pollution control are always advantageous over conventional command and control procedures. Such measures could also promote technological innovation and improvement, resulting in pollution reduction at a comparatively lower cost. However, punitive measures should also be implemented	Future Perspectives and Way forward:<br><br>1) Developing the link between the people and environment: A possible way to overcome the lack of adequate implementation and political will in Pakistan is to develop a proper feedback mechanism to gauge the success and failures of policy options, as well as for identifying possible weaknesses and opportunities.<br><br>2) Coordination between industrial, the public and policy institutions: In the absence of Government legislation, linkages	Poor indoor air quality, due to biomass fuel usage, needs urgent interventions, and it should be locally acceptable and viable. In particular, it should be economical and consider the role of women in the rural energy system and factors responsible for fuel choice decisions. In the first instance, a public awareness campaign regarding the health effects of indoor air pollution should be instigated followed by suitable community-based interventions. Schools and basic health	The quality of life in cities may be greatly improved by switching priorities around and providing for people's most fundamental needs. It suggests controlling population expansion by constructing low- to moderate-energy-consumption, low-travel-need neighborhoods (Rehman et al. 2022). In order to improve the health of communities as a whole, it is important to promote policies that have a less effect on the environment, use less energy, and have a smaller carbon footprint.

ID	Aziz 2006	Hussain 2019	Mir 2024	Mir 2016	Anwar 2021	Anjum 2021	Colbeck 2010	Ahmed 2023
	hour is 200 Î¼g/m3. 3) Nitrogen dioxide: Most countries in the Region have standards for 1-hour and 24-hour concentrations. To keep in line with the standards enforced in these countries, the following guidelines are proposed for NO: 1-hour average = 300 Î¼g/m3 24-hour average = 150 Î¼g/m3. 4) Sulfur dioxide: As SO2 may also exist in particulate form, WHO and many other countries have also specified guidelines on an annual average basis, in addition to 1-hour and 24-	to cope with and diminish those impacts. The formation of an agency with coordination of all these sectors is suggested. The agency will be spearheaded by Reforms and Development Commission and will include representation from various concerning departments with joint operation systems and meeting arrangements. The objective of this agency would be to cooperate together with all departments in formulating and initiating development plans, tasks, policies, and strategies against climate change. The	emission reductions. Promoting fuel switching and clean cooking and heating programs, particularly addressing small wood-burning stoves and fireplaces. Expanding emission standards to cover off-road vehicles, construction equipment and agricultural machinery. Implementing an effective electric vehicle (EV) policy and expanding electricity-based mass transit in major urban centers. Public awareness campaigns and stakeholder engagement.		alongside incentives to ensure the highest levels of environmental protection and pollution mitigation. When explicitly taking air pollution into context, climate policies, complemented with air pollution control policies and energy policies, can reduce air pollution and its pertinent health impacts.<br><br>2) Increasing tree canopies: Trees canopies could also be used as a complementary solution for reducing air pollutants. By lowering air temperature in the surroundings and reducing buildings'	between private and public sectors were weak, and academic institutions can potentially fill the gaps that exist from a lack of coordination and poor communication. There are also possible benefits from an Academic-Industrial linkage in Pakistan. These benefits include the development of R&D, better communication, and chances for innovation in the industrial and academic sectors of Pakistan.<br><br>3) Technological innovations: The present data gaps need to be filled as quickly as possible	units, along with a general media campaign, can provide an avenue to spread the knowledge of indoor air pollution due to biomass fuel usage and indoor smoking across the country. Due to the current socioeconomic conditions in the country, development and adoption of improved cooking stoves for the population at large would be the most suitable choice. In addition to improved stoves, there is potential for using biogas as a rural energy source. There is a need for an integrated approach and the financial	Envision a community that has its own neighborhood board, schools, parks, and low-rise but high-density housing, as well as roads and roadways that have bike lanes and walkways running parallel to them. In order for urbanization in Pakistan to succeed, demand management is essential (United Nations, Department of Economic and Social Affairs 2018).

ID	Aziz 2006	Hussain 2019	Mir 2024	Mir 2016	Anwar 2021	Anjum 2021	Colbeck 2010	Ahmed 2023
	hour averages. The suggested guidelines for SO2 levels for Pakistan are: 1-hour = 350 Î¼g/m3 24-hour = 125 Î¼g/m3 Annual = 80 Î¼g/m3. 5) Lead: Under the present conditions, it would be appropriate to adopt WHO guidelines for Pakistan for lead of an annual average of 1.0 Î¼g/m3. 6) Total suspended particulate matter: The following guidelines for TSP levels are proposed, in line with the values adopted by countries with conditions similar to Pakistan: 24-hour = 500	agency's role in the country would be to cooperate and coordinate with several other institutions such as non-governmental organizations, non-profit institutions, entrepreneurs, philanthropists, specific communities, and general public while formulating, initiating, and exercising plans and policies regarding climate change mitigation and adaption. The policies have to be formulated at every level including national, provincial, divisional, and local level, with continual			energy use, trees could contribute to mitigating air pollutants. The removal rate of air pollutants by trees varies depending upon several factors like leaf area index, local weather conditions, percentage of evergreen trees, pollution concentration, and growing season length. Trees could also moderate the heat-island effect caused by pavement materials and glass buildings. Moreover, regions with dense tree covers could recycle water through the evapotranspirati on process more effectively than areas where vegetation cover	through the development of low-cost continuous monitoring systems or through supplementing ground-based monitoring with satellite observations to obtain a complete picture. Wireless sensor networks (WSNs) are robust, cost, and energy efficient solutions to various agricultural issues in Pakistan because of their versatile nature. The development of low-cost gas sensors embedded in a wireless sensor network could be a more suitable option for effective air	support by the Government, and the involvement of various community-based organizations is vital for the success. Moreover, access to modern cooking fuels (natural gas, LPG) should be enhanced. Strict legislation on smoking in confined public places should be implemented. General public awareness about role of indoor smoking in the deterioration of indoor air quality and hazardous health effects of smoking along with practical support to quit should be provided at basic health	

ID	Aziz 2006	Hussain 2019	Mir 2024	Mir 2016	Anwar 2021	Anjum 2021	Colbeck 2010	Ahmed 2023
	Î¼g/m (excluding duststorm days) Annual = 300 Î¼g/m. 7) Particulate matter: Many countries in the Regions employ PM standard. The following values for PM levels are suggested: 24-hour average = 200 Î¼g/m3 (excluding duststorm days) Annual average = 120 Î¼g/m3.	assessment of policies to ensure accountability and effectiveness of policies. The formulation of policies should follow a bottom-to-top flow, with local and divisional governments enforcing the policies regarding transportation, industrialization, energy production and consumption, urbanization, waste management, and agricultural activities, while national government periodically assessing the outcomes of regulations and federal government as the highest authority.			is sparse.<br><br>3) Improving air quality reporting systems: Despite the alarming situation, air quality monitoring is still done only in urban areas where hazardous/ visible air pollution can be clearly seen. This is ascribed to the lack of a skilled workforce and advanced analytical laboratories. Environmental drones (E-Drones) could be a way forward and a solution to this problem. E-Drones are autonomously programmed drones used for pollution detection and	pollution monitoring in Pakistan.<br><br>4)Raising awareness: The government may provide subsidies and introduce incentives for opting to clean production technologies. Introduction of self-monitoring and reporting technology (SMART) could be an essential initiative towards the decrease in industrial pollution. Several outreach programs could be initiated in order to raise awareness among masses, especially targeting the school children, and air pollution as part of the curriculum at a	units in the country.	

ID	Aziz 2006	Hussain 2019	Mir 2024	Mir 2016	Anwar 2021	Anjum 2021	Colbeck 2010	Ahmed 2023
		The main objective of climate change policies should be to aid in enriching the population with comprehensive socio-scientific skillsets, advanced technologies, renewable energy resources, and knowledge regarding sustainable processes for climate change mitigation and adaptation. These policies should aim at decreasing excessive usage of environment degrading products and processes through delegated responsibility to the officials at divisional level for effective			monitoring at specified altitudes in any geographic range. E-Drones can create air quality health index (AQHI) maps of the regions within their monitoring range and could assist with long-term data analysis.<br><br>4) Waste-to-energy (WtE): A practical solution to the air pollution challenge could be the development and implementation of WtE technologies. For instance, municipal solid wastes incineration can reduce its mass by up to 70%, reduce GHGs emissions, and reduce	very early stage could be beneficial. Air pollution caused by agricultural burning can be avoided by introducing; strict legislation to ban the open burning at all scales, especially during the smog season, subsidies for farmers to opt for other methods for safe disposal of crop residue such as composting and biogas production.<br><br>5) Role of academia: Academic institutions have a vast potential that can be used to help bridge the gaps between policy institutions, government bodies,		

ID	Aziz 2006	Hussain 2019	Mir 2024	Mir 2016	Anwar 2021	Anjum 2021	Colbeck 2010	Ahmed 2023
		chain of command.<br><br>Transparency in decision-making process and formulation of formal policy with clear objectives, allocation of resources, and strategic targets can improve the implementation tenure of regulations for a long time.			landfilling. During the incineration process, the municipal waste's organic content is combusted under controlled conditions, producing renewable energy that could be substitute fossil fuels. However, inevitable byproducts are also formed during the process, such as hazardous gases, pollutant particles, and fly ash. Therefore, it is essential to ensure that state-of-the-art technologies will be utilized to minimize incineration's negative impacts on the environment. Anaerobic digestion of	industries, and the public. In order to promote environmentally sustainable development (ESD) in Pakistan, the role of the educational institutes must be expanded so that they can reach all the critical stakeholders of ESD, which include governmental agencies, industry, NGOs, and the public.Wherein academia has the role of interpreting the primary science involved in the processes, providing updates about recent developments to GOs, providing ground information about the		

ID	Aziz 2006	Hussain 2019	Mir 2024	Mir 2016	Anwar 2021	Anjum 2021	Colbeck 2010	Ahmed 2023
					organic waste for biogas production is another major WtE approach that could lead to the generation of biomethane as a replacement for natural gas. Conversion of waste oils of plant and animal origin into biodiesel to partially replace diesel consumption would be another WtE solution to be implemented. Despite the advantageous features of WtE systems, it is still critical to look into their sustainability features using advanced sustainability assessment tools.<br><br>5) Promoting low-sulfur fuel:	observed phenomenon to the NGOs to work upon, prepare the database, provide timely feedback to the policymakers about the effectiveness of devised policies and act as a bridge to ensure effective communication between the public and other relevant stakeholders.<br><br>Policy Recommendations:<br>1) Reducing the price and promoting the use of efficient fuel sources in the industrial and power generation sector, and restraining the use of highly polluting plastic and coal-based		

ID	Aziz 2006	Hussain 2019	Mir 2024	Mir 2016	Anwar 2021	Anjum 2021	Colbeck 2010	Ahmed 2023
					Health impacts and other environmental constraints have led to limitations on the sulfur level present in crude oil-derived products globally, necessitating desulfurization. During the recent three decades, numerous desulfurization techniques have been introduced, including oxidation-extraction desulfurization, bio-desulfurization, adsorptive desulfurization, oxidative desulfurization, and hydrodesulfurization. Hydrodesulfurization is the most common method	fuels.<br>2) Enforcing better technological standards to reduce pollution in the transportation and industrial sectors. This includes the use of catalytic converters, scrubbers, and cleaner fuels.<br>3) Extensive monitoring of air pollutants and identification of their sources by developing a consistent and continuous monitoring network throughout Pakistan to fill the present knowledge gaps.<br>4) Revision of current national and provincial air quality standards to expand the number of		

ID	Aziz 2006	Hussain 2019	Mir 2024	Mir 2016	Anwar 2021	Anjum 2021	Colbeck 2010	Ahmed 2023
					employed in refineries.<br><br>6) Providing alternatives for stubble burning: Every year burning of stubble is carried out from mid-April to mid-May after harvesting the wheat crop and from October to November after harvesting rice crop in different regions in Pakistan and India. Open burning releases various pollutants such as hydrocarbons and aerosols into the air. Any alternatives to open field burning of crop residues can substantially contribute to air pollution mitigation. The conversion of rice straw into	outdoor and industrial pollutants and development of indoor air quality standards in Pakistan.<br>5) Promoting the use of public transportation and to discourage/ban the use of poorly maintained/ polluting vehicles in the country.<br>6) Promoting appropriate public discourse and disclosure schemes that require industries to report their emission and policymakers to accept appropriate feedback during the policy process.<br>7) Development of the role of academia and		

ID	Aziz 2006	Hussain 2019	Mir 2024	Mir 2016	Anwar 2021	Anjum 2021	Colbeck 2010	Ahmed 2023
					bioethanol could be regarded as alternatives to burning; this rice straw management technique could be costly, though. Soil fertility can be increased through in situ utilization of stubble. It is a good source of sulfur and potassium and contains nitrogen and phosphorus as well. The stubble co-composting with the poultry manure has also been reported in recent years.	encouragement of R&D culture in industries as well.<br>8) To adopt better air pollution management practices, development of emission inventories and source apportionment of pollutants to devise cost-effective and efficient air pollution abatement strategies.		
**Other recommendations**	-	The implications of these policies will be apparent through strong	To enhance the accuracy of BC emissions estimation, future research	In terms of future research, a cost-effective strategy could be developed	-	Transboundary air pollution needs to be addressed between	The work carried out by various governmental organizations	Further investigation of land use, land cover, settlement

ID	Aziz 2006	Hussain 2019	Mir 2024	Mir 2016	Anwar 2021	Anjum 2021	Colbeck 2010	Ahmed 2023
		support from the population, longer continual period of the policies, and general change towards sustainable behaviors from the population of the country.	efforts should prioritize a) developing more precise emission factors for key sources, using locally measured data; b) enhancing the collection of activity data for all BC emission sources; and c) conducting studies to better understand the actual performance of control technologies. International collaborations and support, integrating localized data and expertise related to emission sources and their associated factors.	with the help of Pakistan Integrated Energy Model (Pak-IEM) and the optimization mode of the GAINS model. End-of-pipe emission control measures include selective catalytic reduction for lowering NOx emissions, desulfurization of flue gases for lowering SO2 emissions, use of high-efficiency devices for regulating PM emissions from various industrial processes, and innovative emission control measures to control emissions from vehicles (i.e., Euro-VI in EU), among others.		neighbors (countries) and, if correctly approached, can lead to provide an opportunity to initiate communication, exchange of expertise, and reforms between neighboring states.	(The National Institute of Silicon Technology, Pakistan Council of Scientific and Industrial Research, Pakistan Council of Appropriate Technology) on renewable energy resources needs consideration and marketing.	expansion analysis, and urban growth modeling using dense satellite dataset and satellite imaging, to capture decadal and seasonal timeframes, is needed. To gain better understanding of heat island effect and global warming, temperature and humidity readings of different union councils should be considered in future research. This can also be used for other global temperature and rainfall analysis within the context of urbanization and climate change impacts. More research into local adaptive capacity for the

ID	Aziz 2006	Hussain 2019	Mir 2024	Mir 2016	Anwar 2021	Anjum 2021	Colbeck 2010	Ahmed 2023
								entire country would enrich the assessment of localized analysis. Energy-mix in Pakistan is not favorable for clean and green environment, but the government can provide subsidy on solar panels and provide it to the most vulnerable parts of these societies at lower price, which will not only be environmentally friendly but also it will lessen the energy costs of the poor and marginalized people. Cheap and environment friendly transportation in cities should be the topmost priority of the local and

ID	Aziz 2006	Hussain 2019	Mir 2024	Mir 2016	Anwar 2021	Anjum 2021	Colbeck 2010	Ahmed 2023
								national governing bodies. For this, mass-transit authorities of each city should further enhance routs of metro busses and trains throughout the city. Single vehicle commuters of rich class can be brought into the metro-busses and trains by offering segregation in this mass-transit system in the form of business class where higher fares can be charged, and this will allow the authorities to offer even lower fares for the economy class commuters.
**Additional information**	-	Pakistan has a potential to become one of the highest	Pakistan, a country heavily reliant on fossil fuels, faces	The Asian Development Bank (ADB) supported the	-	According to the institute for health metrics and evaluation,	Use of biomass fuel as an energy source is the biggest	-

ID	Aziz 2006	Hussain 2019	Mir 2024	Mir 2016	Anwar 2021	Anjum 2021	Colbeck 2010	Ahmed 2023
		emitters of greenhouse gases by 2030 due to its swift urbanization, unrelenting energy demands, increasing transportation, and improper waste management. In addition to said societal change, Pakistan's adaptive capacity is hindered by scarcity of physical and financial resources, whereas prevailing poverty rate is also a salient factor.	significant air pollution challenges, including high levels of PM2.5 emissions. These emissions stem primarily from the combustion of fossil fuels and biomass burning. Fossil fuel-related emissions primarily originate from coal-based power plants and diesel vehicle tailpipes, while biomass burning is associated with traditional cooking stoves and the open burning of agricultural residue. These emissions contribute to high ambient levels of PM2.5 in Pakistan, which in turn are	Planning Commission of Pakistan to help the Government of Pakistan (GoP) in establishing an integrated energy model, entitled "Pakistan Integrated Energy Model" (Pak-IEM). Pak-IEM uses the The Integrated MARKAL/EFOM System (TIMES) model framework that allows the assessment of policies aimed at meeting the future energy demands of the country in an optimal way.<br><br>In terms of health impacts, the GAINS results show that the annual mean concentration of fine particulate		air pollution was ranked as the highest environmental/ occupational factor for most deaths and disabilities in Pakistan during 2017, and the 5th highest factor overall.<br><br>The most cited source of air pollution in Pakistan, according to the reviewed literature were vehicular emissions followed by thermal power generation, industry, biomass burning, and transboundary.<br><br>Higher concentrations of air pollutants during winters compared to summer, most likely due to the	contributor to poor indoor air quality followed by smoking.<br><br>Indoor air pollution is a significant economic burden in Pakistan and annually costs 1% of GDP. Women and children are the most exposed proportion of the population due to amount of time spent near the stove or as passive smokers in the indoor environment.<br><br>Example of behavior change: Having the stove at waist height would reduce the need to lean over the fire and hence reduce direct exposure to smoke.	

ID	Aziz 2006	Hussain 2019	Mir 2024	Mir 2016	Anwar 2021	Anjum 2021	Colbeck 2010	Ahmed 2023
			linked to approximately 114,000 annual deaths, with BC (soot) constituting a significant fraction of PM2.5.<br><br>Residential combustion is the major BC emissions source, contribÂuting 58.5% of total emissions. Transportation, industrial combustion and processes, MSW open burning, and agricultural residue burning were also significant contributors.<br><br>The brick industry poses a major air pollution challenge, especially in urban areas with high	matter (PM2.5), which already surpasses the acceptable annual level of the WHO of 10 Î¼g/m3 (WHO 2006) almost throughout Pakistan, typically reaches in the Punjab region in the range of 50 to 100 Î¼g/m3 in 2007. By 2030, such levels would be extended over most of Pakistan, while in some parts of the Punjab region, concentrations would increase to more than 150 Î¼g/m3. Consequently, health impacts resulting from increased air pollution would increase. For outdoor pollution, loss in statistical life		phenomenon of temperature inversion and increased burning of biomass for heating purposes.<br><br>The high particulate matter concentrations during the pre-monsoon periods are most likely a result of heavy crop residue-burnings and frequent dust storms that occur during this period. The particulate concentrations during the monsoon season are comparatively lower than the rest of the year, most likely a result of the frequent rains and higher atmospheric mixing ratios.	The Fuel Efficient Cooking Technologies project: It resulted in the production and dissemination of some 40,000 stoves in 1990. A later program, on fuel-saving technologies, provided incentives to NGOs and community-based organizations for its implementation. However, these stoves were not considered economical for poor families. The stoves were far better in terms of heating capacity, wood saving, cooking efficiency, and smoke reduction as compared to their traditional counterparts.	

ID	Aziz 2006	Hussain 2019	Mir 2024	Mir 2016	Anwar 2021	Anjum 2021	Colbeck 2010	Ahmed 2023
			concentrations of brick kilns. The combustion of coal, wood, or biomass fuels in this process emits harmful pollutants like black carbon, PM (particulate matter), SO2, NOx, and CO, contributing to deteriorating air quality, smog formation, and adverse impacts on human health and the environment.<br><br>The United Nations Environment Programme (UNEP) has launched the Climate and Clean Air Coalition (CCAC) with a specific focus on reducing short-lived climate pollutant (SLCP)	expectancy is calculated to increase from 30 to 60 months in 2007 up to 60â€“100 months in 2030 for the Pakistani population on an average and to exceed to 100 months in few parts of Pakistan. The total number of years of life lost due to outdoor air pollution would increase by a factor of 2 from 4 years lost/year in 2007 to 8 years lost/year in 2030.<br><br>The maximum use of advanced end-of pipe emission control measures in large combustion sources can result in significant environmental,		The post-monsoon and winter seasons exhibited the highest particulate matter concentrations compared to the rest of the seasons. These high concentrations are most likely a result of increased human activities, like crop residue burning across both Pakistan and India during relatively stable atmospheric conditions, i.e., lower boundary layer heights and lower mixing rate.<br><br>A very interesting feature is observed with air quality conditions during night-	Building and Construction Improvement Program (BACIP): Most interventions have focused on northern areas of Pakistan due to the degradation of natural resources. BACIP, established in 1997, has installed over 17,000 energy-efficient and living condition improvement products in various households, benefiting nearly 70,000 people across 125 villages. These include fuel-efficient smoke-free cooking stoves with chimneys, as well as wall and floor insulation, and	

ID	Aziz 2006	Hussain 2019	Mir 2024	Mir 2016	Anwar 2021	Anjum 2021	Colbeck 2010	Ahmed 2023
			emissions (including BC) in developing nations like Pakistan. At the global level the World Health Organization (WHO) has developed air quality guidelines, including limits on annual PM2.5 concentrations, to safeguard public health. At the regional level, the South Asian Association for Regional Cooperation (SAARC) has devised a strategy through the "SAARC Convention on Cooperation on Environment" to combat the health and climate impacts of air pollution. Clean Air Asia (CAA) has	health, and societal benefits for Pakistan because of improvement in ambient air quality. It is estimated that negative health impacts from air pollution could be reduced by 56% in 2030 by applying such advanced emission control technology to all large sources in Pakistan.		time (7 pm to 5 am), which show several inÂstances of higher pollution concentrations than the daytime conditions, i.e., nights exhibited higher instances of hazardous and unhealthy categories as compared to days which exhibited more moderat and less hazardous categories. This was most likely a result of reduced atmospheric mixing and stable night-time conditions, which can lead to higher pollutant accumulation in the atmosphere. Another possible reason is that most of the heavy traffic is only allowed to enter the city	roof hatch windows to reduce dust particles and improve indoor heating. In 2003, a BACIP, with support from local government, was commenced in Sindh province. A similar participatory research and implementation process to that in the North has allowed for easy replication, and new products have been designed that match cultural and climatic requirements.<br><br>Biogas program: Apart from improved cooking stoves, the Government of Pakistan started a comprehensive biogas program	

ID	Aziz 2006	Hussain 2019	Mir 2024	Mir 2016	Anwar 2021	Anjum 2021	Colbeck 2010	Ahmed 2023
			actively worked across Asia to mitigate SLCP (including BC) emissions. The European Union (EU) has implemented a range of comprehensive measures targeting BC emissions, encompassing regulations on vehicle emissions, industrial processes, and residential heating. The World Bank has established the Global Gas Flaring Reduction (GGFR) Partnership to address BC emissions resulting from natural gas flaring. The Global Green Freight Action Plan, a collaborative			during night-time (after 10 pm). While another reason might be the growing trend of increased industrial activities using dirty fuel (such as trash burning, rubber bricks, etc.) taking place at night-time in Lahore to avoid monitoring by the regulatory authorities.<br><br>Pakistan's vehicular mix has a sizable portion composed of diesel consuming vehicles, and vehicles are old, poorly maintained, smoky, and employ outdated engines. Average annual growth rate for on-road vehicles	in 1974 and had commissioned 4,550 plants by 1990. The program was developed in three phases. Initially, the government installed 100 units, and in the second phase, the cost was shared between the Government and beneficiaries. In third phase, only beneficiaries bore the cost. Due to withdrawal of Government financial support, the program did not progress. Although these pilot projects showed promising results, they were at a small scale and lacked the coordination among all the stakeholders.	

ID	Aziz 2006	Hussain 2019	Mir 2024	Mir 2016	Anwar 2021	Anjum 2021	Colbeck 2010	Ahmed 2023
			effort among international organizations, aims to reduce BC emissions in the transportation sector by promoting fuel-efficient vehicles and improved logistics practices.			was more than 8.5% and when comparing these vehicles to standards in the US found that an average vehicle in Pakistan produces 25, 20, 8 and 3.6 times higher carbon, Hydrocarbons, lead and NOx per kilometer respectively, when compared with a vehicle from the US.<br><br>Steel, sugar, cement, and fertilizer industries have been labeled as major contributors to poor air quality because of their use of high sulphur containing furnace oil and the production of particulate pollution. Some medium and		

ID	Aziz 2006	Hussain 2019	Mir 2024	Mir 2016	Anwar 2021	Anjum 2021	Colbeck 2010	Ahmed 2023
						small-scale industries such as brick kilns, steel recycling, and plastic molding industries have also been linked with poor urban air quality mostly because of their use of waste fuels such as old tires, paper, wood, textile and biomass and their use of old and worn out equipment such as boilers and generators.<br><br>Solid waste collected in communal bins are usually set on fire to reduce the volume and has become a common occurrence associated with vast amounts of air pollution.		
